# Supplementary material for: On the Significance of the Terminal Location of Prion-Forming Regions of Yeast Proteins
Source: Int J Mol Sci. 2025 Feb 14;26(4):1637. doi: 10.3390/ijms26041637 (PMC11855515; doi:10.3390/ijms26041637)
Supplement: Supplementary file 1 [file ijms-26-01637-s001.zip › ijms-3383742-supplementary.pptx]

## Slide 1
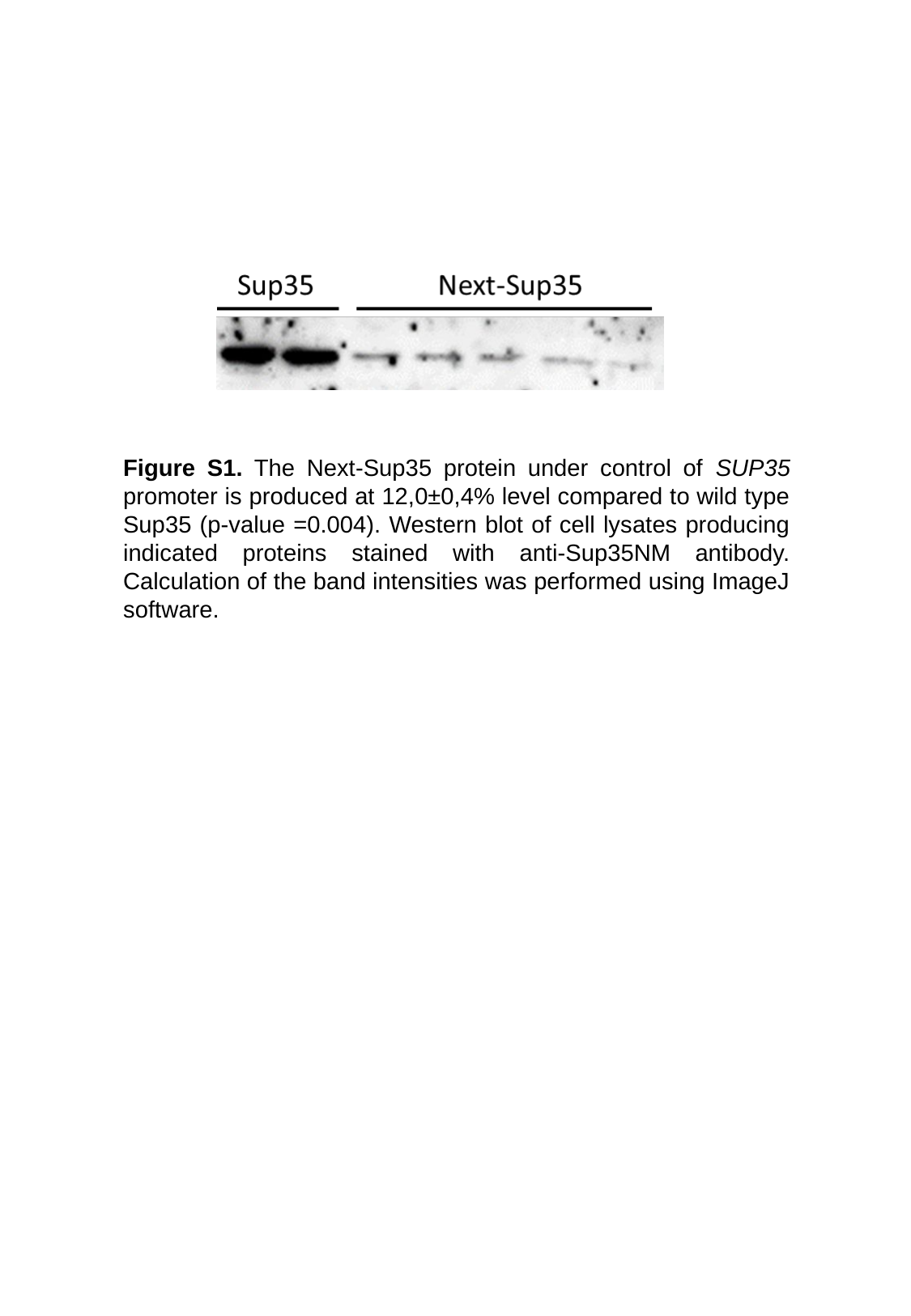

Figure S1. The Next-Sup35 protein under control of SUP35 promoter is produced at 12,0±0,4% level compared to wild type Sup35 (p-value =0.004). Western blot of cell lysates producing indicated proteins stained with anti-Sup35NM antibody. Calculation of the band intensities was performed using ImageJ software.

## Slide 2
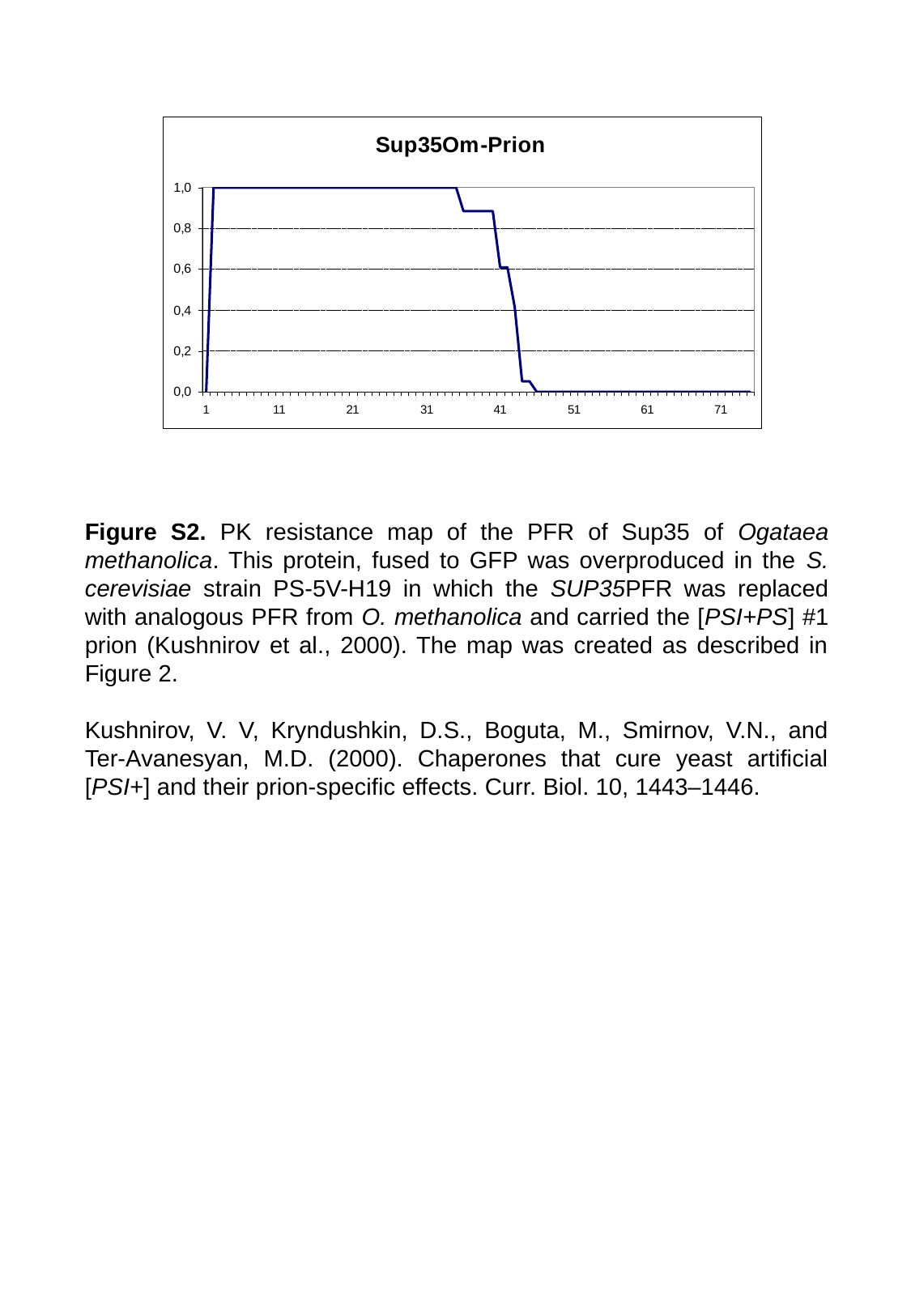

Figure S2. PK resistance map of the PFR of Sup35 of Ogataea methanolica. This protein, fused to GFP was overproduced in the S. cerevisiae strain PS-5V-H19 in which the SUP35PFR was replaced with analogous PFR from O. methanolica and carried the [PSI+PS] #1 prion (Kushnirov et al., 2000). The map was created as described in Figure 2.
Kushnirov, V. V, Kryndushkin, D.S., Boguta, M., Smirnov, V.N., and Ter-Avanesyan, M.D. (2000). Chaperones that cure yeast artificial [PSI+] and their prion-specific effects. Curr. Biol. 10, 1443–1446.

## Slide 3
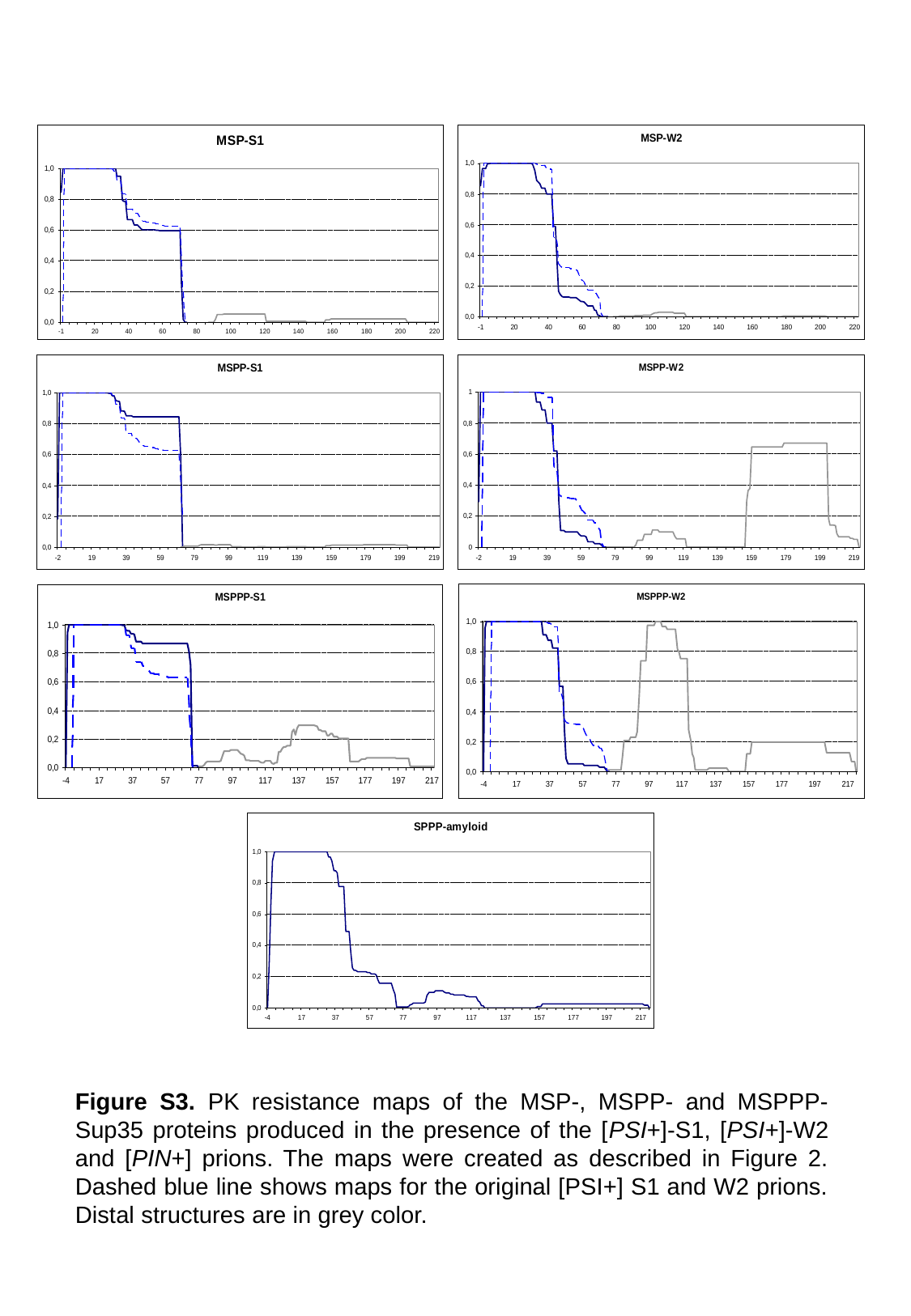

Figure S3. PK resistance maps of the MSP-, MSPP- and MSPPP-Sup35 proteins produced in the presence of the [PSI+]-S1, [PSI+]-W2 and [PIN+] prions. The maps were created as described in Figure 2. Dashed blue line shows maps for the original [PSI+] S1 and W2 prions. Distal structures are in grey color.

## Slide 4
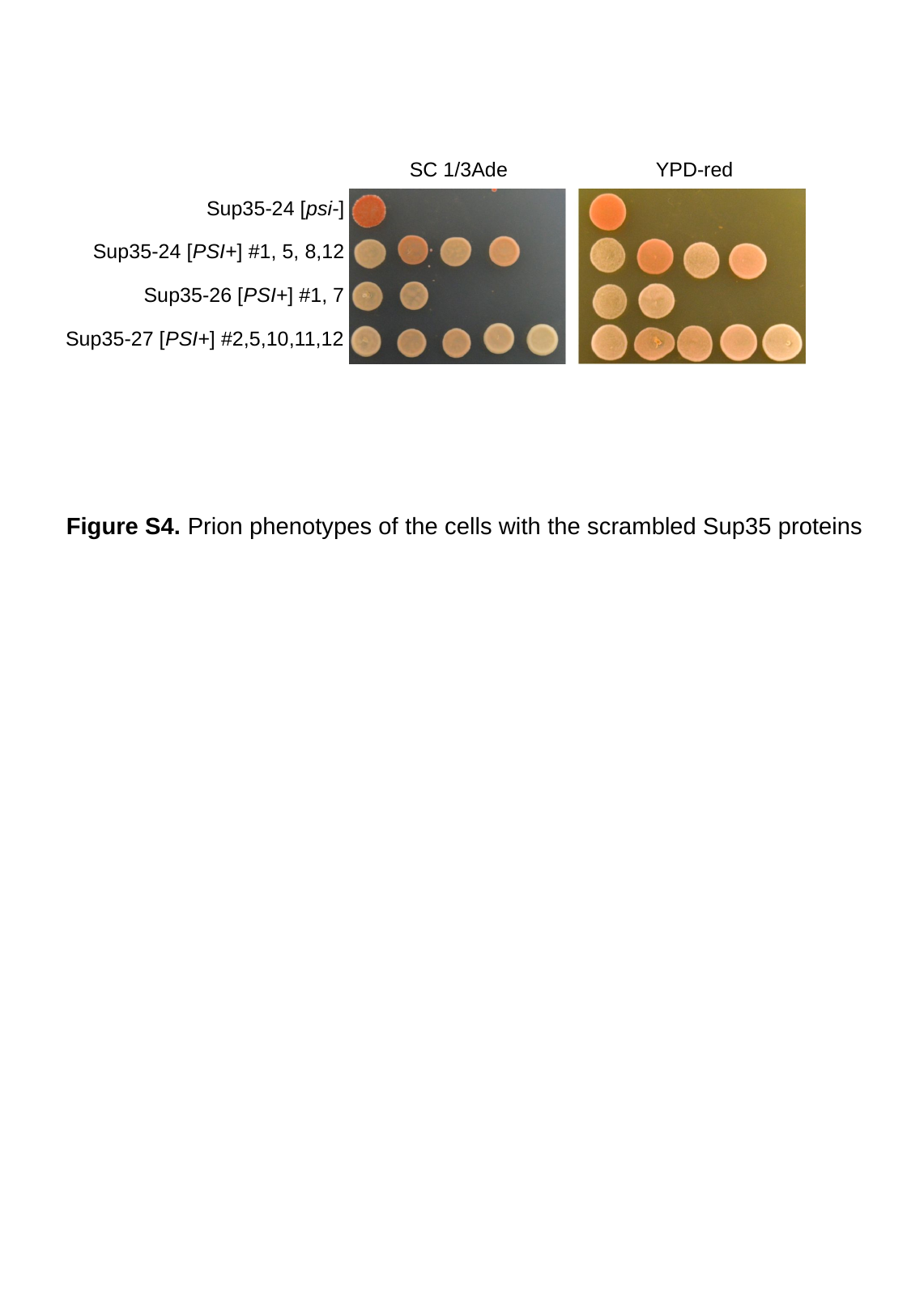

SC 1/3Ade
YPD-red
Sup35-24 [psi-]
Sup35-24 [PSI+] #1, 5, 8,12
Sup35-26 [PSI+] #1, 7
Sup35-27 [PSI+] #2,5,10,11,12
Figure S4. Prion phenotypes of the cells with the scrambled Sup35 proteins

## Slide 5
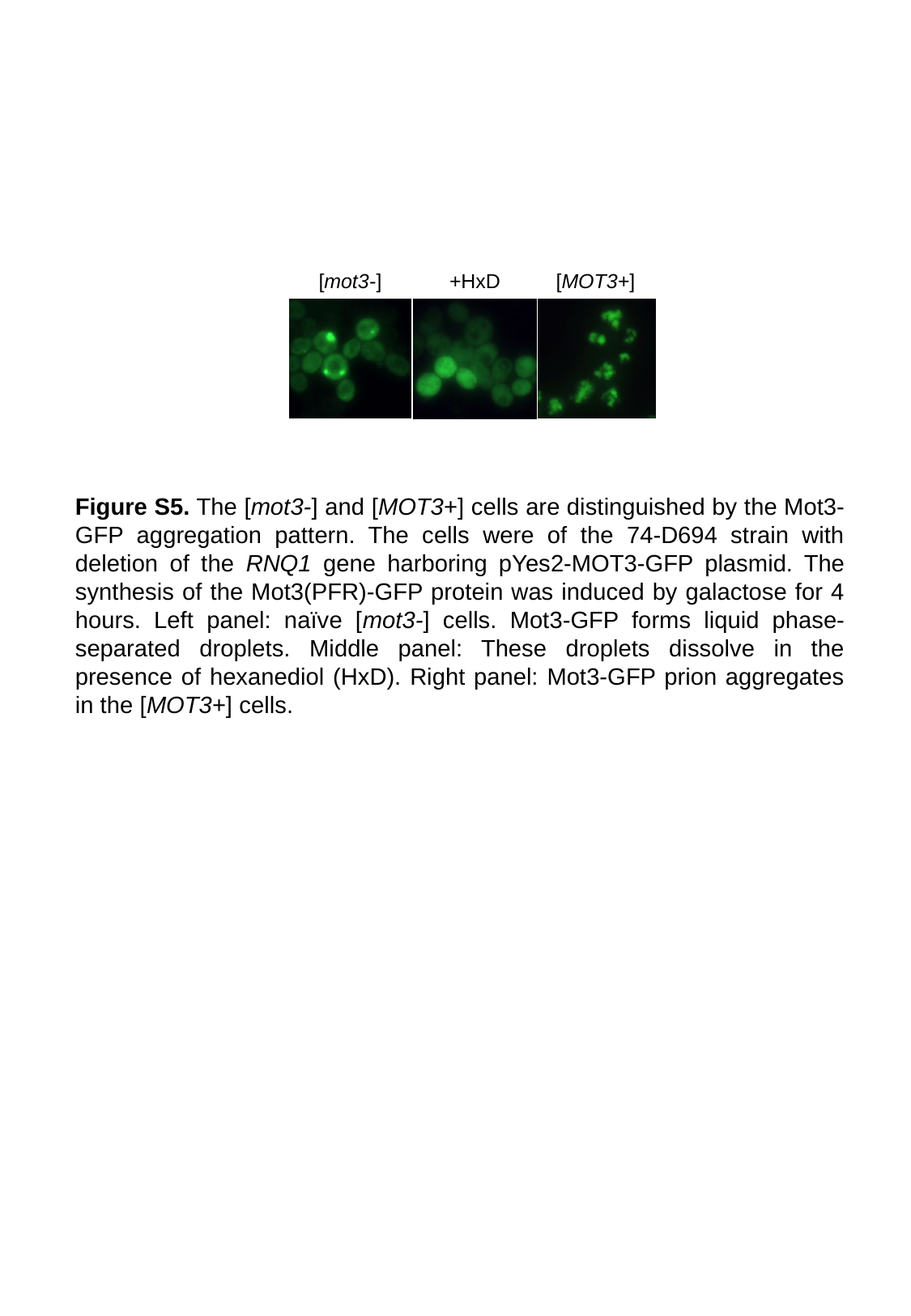

+HxD
[mot3-]
[MOT3+]
Figure S5. The [mot3-] and [MOT3+] cells are distinguished by the Mot3-GFP aggregation pattern. The cells were of the 74-D694 strain with deletion of the RNQ1 gene harboring pYes2-MOT3-GFP plasmid. The synthesis of the Mot3(PFR)-GFP protein was induced by galactose for 4 hours. Left panel: naïve [mot3-] cells. Mot3-GFP forms liquid phase-separated droplets. Middle panel: These droplets dissolve in the presence of hexanediol (HxD). Right panel: Mot3-GFP prion aggregates in the [MOT3+] cells.

## Slide 6
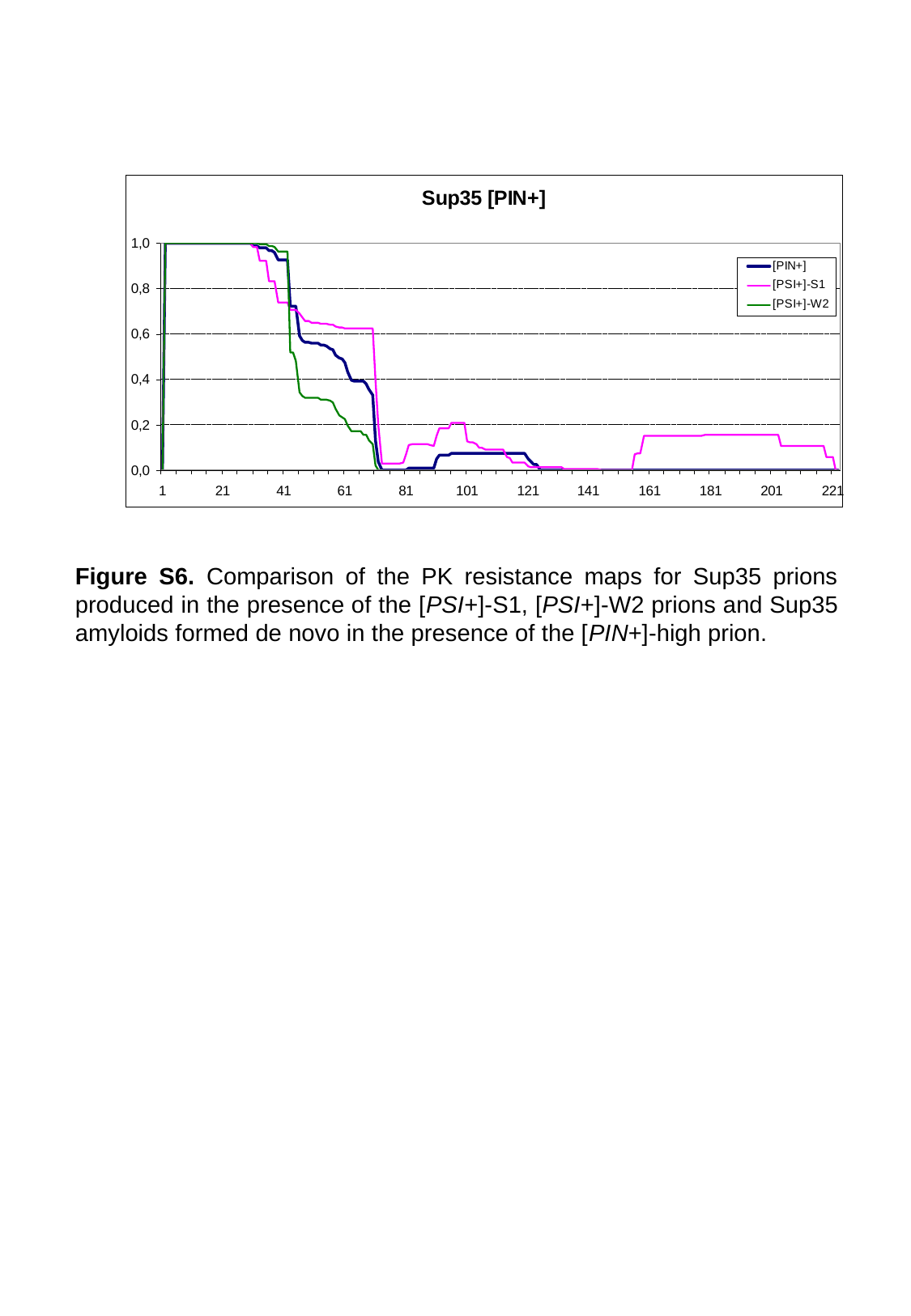

Figure S6. Comparison of the PK resistance maps for Sup35 prions produced in the presence of the [PSI+]-S1, [PSI+]-W2 prions and Sup35 amyloids formed de novo in the presence of the [PIN+]-high prion.

## Slide 7
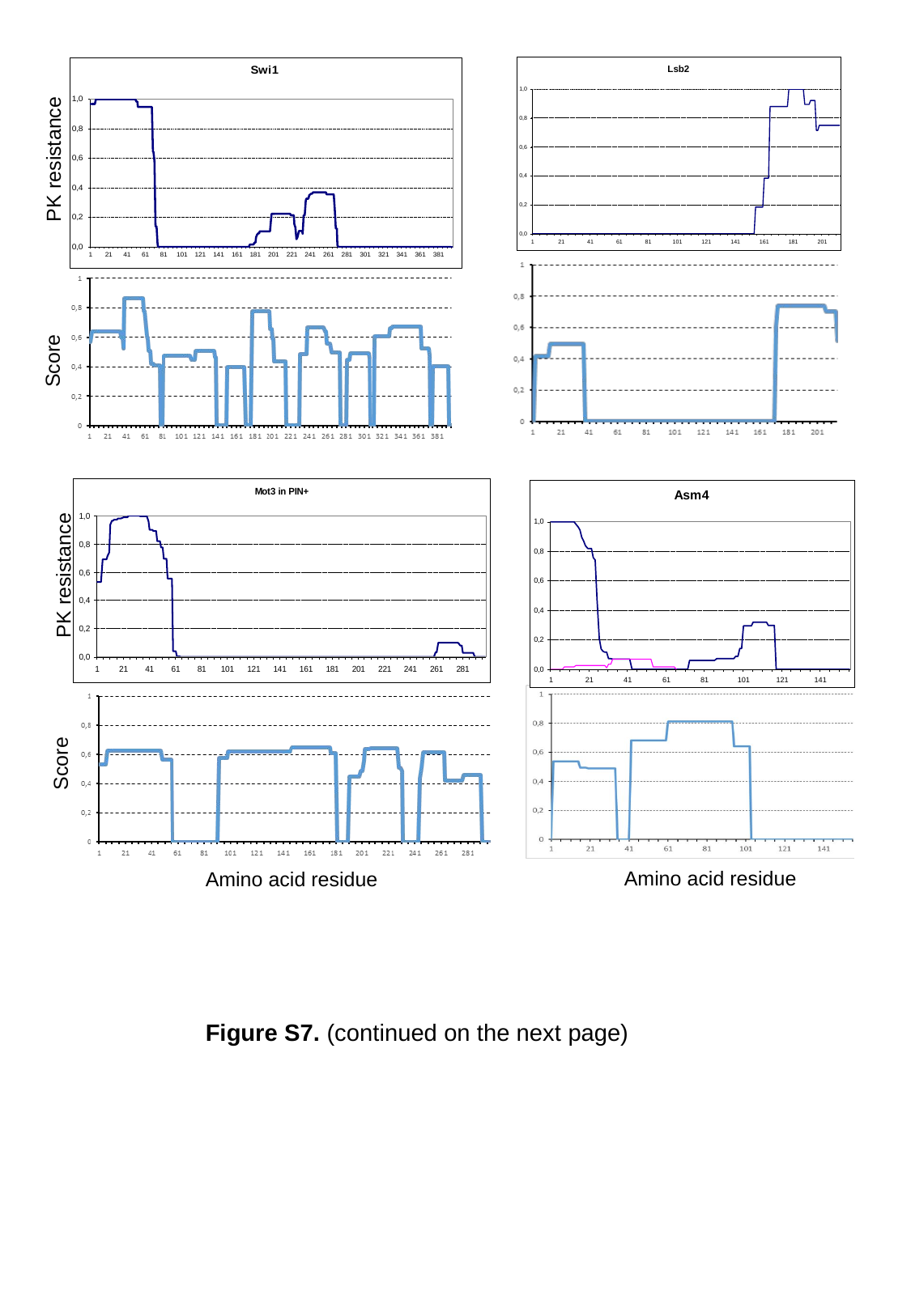

PK resistance
Score
PK resistance
Score
Amino acid residue
Amino acid residue
Figure S7. (continued on the next page)

## Slide 8
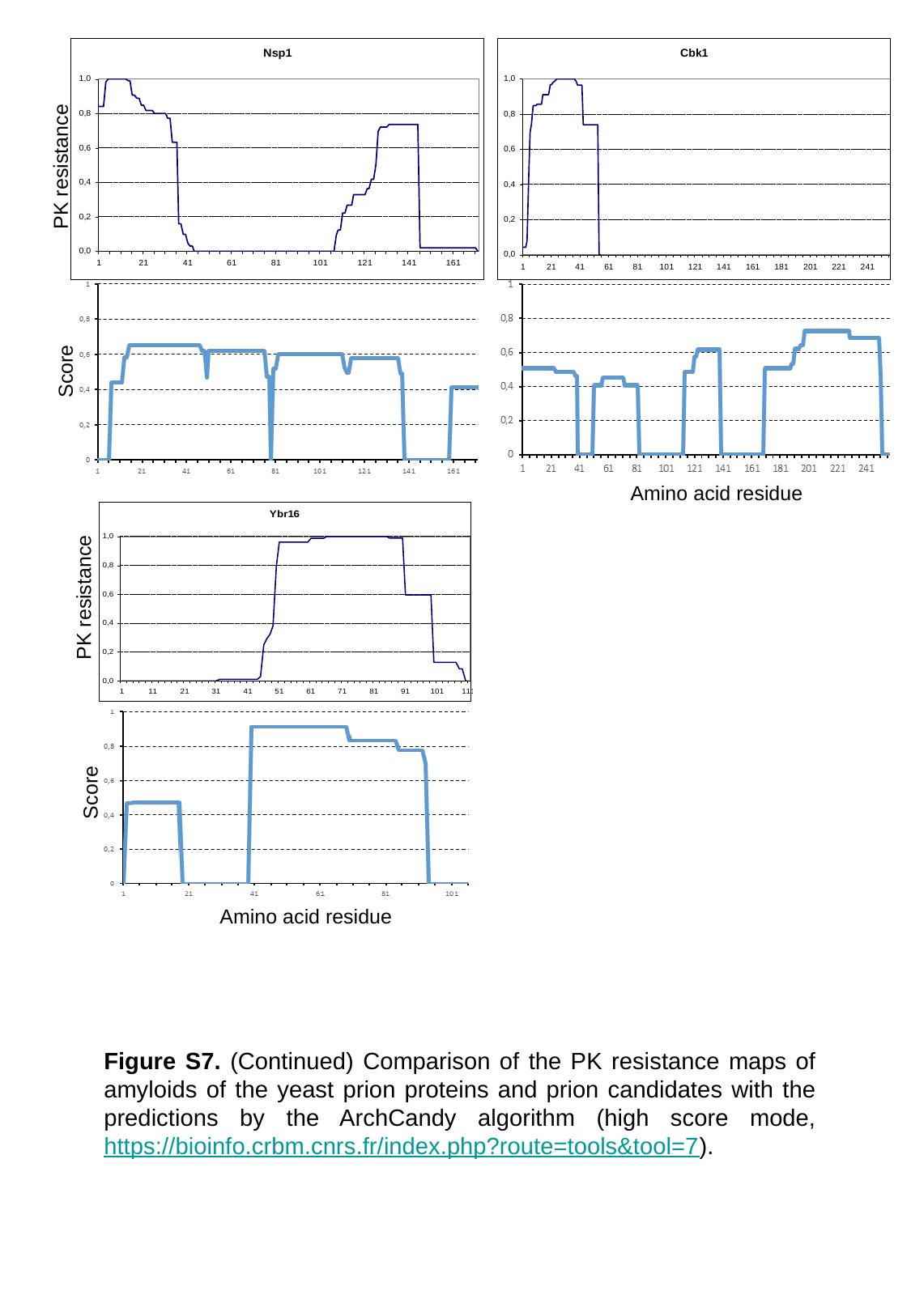

PK resistance
Score
Amino acid residue
PK resistance
Score
Amino acid residue
Figure S7. (Continued) Comparison of the PK resistance maps of amyloids of the yeast prion proteins and prion candidates with the predictions by the ArchCandy algorithm (high score mode, https://bioinfo.crbm.cnrs.fr/index.php?route=tools&tool=7).
